# Inhalable Spray-Dried Chondroitin Sulphate Microparticles: Effect of Different Solvents on Particle Properties and Drug Activity

**DOI:** 10.3390/polym12020425

**Published:** 2020-02-12

**Authors:** Susana Rodrigues, Ana M. Rosa da Costa, Noelia Flórez-Fernández, María Dolores Torres, Maria Leonor Faleiro, Francesca Buttini, Ana Grenha

**Affiliations:** 1 Centre for Marine Sciences, Faculdade de Ciências e Tecnologia, Universidade do Algarve, 8005-139 Faro, Portugal; susananasus@gmail.com (S.R.); noelia.florez@uvigo.es (N.F.-F.); 2 Centre for Biomedical Research, Universidade do Algarve, 8005-139 Faro, Portugal; mfaleiro@ualg.pt; 3 Algarve Chemistry Research Centre and Department of Chemistry and Pharmacy, Universidade do Algarve, 8005-139 Faro, Portugal; amcosta@ualg.pt; 4 Faculdade de Ciências e Tecnologia, Universidade do Algarve, 8005-139 Faro, Portugal; 5 Department of Chemical Engineering, University of Vigo, Faculty of Sciences, As Lagoas, 32004 Ourense, Spain; matorres@uvigo.es; 6 Food and Drug Department, University of Parma, 43124 Parma, Italy; francesca.buttini@unipr.it

**Keywords:** chondroitin sulphate, isoniazid, inhalable microparticles, rifabutin, solvents, spray-drying

## Abstract

Spray-drying stands as one of the most used techniques to produce inhalable microparticles, but several parameters from both the process and the used materials affect the properties of the resulting microparticles. In this work, we describe the production of drug-loaded chondroitin sulphate microparticles by spray-drying, testing the effect of using different solvents during the process. Full characterisation of the polymer and of the aerodynamic properties of the obtained microparticles are provided envisaging an application in inhalable tuberculosis therapy. The spray-dried microparticles successfully associated two first-line antitubercular drugs (isoniazid and rifabutin) with satisfactory production yield (up to 85%) and drug association efficiency (60%–95%). Ethanol and HCl were tested as co-solvents to aid the solubilisation of rifabutin and microparticles produced with the former generally revealed the best features, presenting a better ability to sustainably release rifabutin. Moreover, these presented aerodynamic properties compatible with deep lung deposition, with an aerodynamic diameter around 4 μm and fine particle fraction of approximately 44%. Finally, it was further demonstrated that the antitubercular activity of the drugs remained unchanged after encapsulation independently of the used solvent.

## 1. Introduction

Polysaccharides have been frequently used as matrix materials of inhalable microparticles aimed at lung drug delivery and spray-drying is a predominant technique applied in their production. Natural materials show the general advantage of having a higher probability to provide biocompatibility and biodegradability, rendering them attractive for pharmaceutical applications. Chondroitin sulphate (ChS) is a natural polymer commonly found on proteoglycans in several tissues, including the lungs [1]. It is composed of alternating sulphated *N*-acetylgalactosamine and glucuronic acid residues, both referred to be recognised by macrophage receptors [2,3], which provides an important advantage in macrophage targeting strategies [4]. Tuberculosis is a respiratory disease characterised by the intracellular accumulation of the infectious agent (*Mycobacterium tuberculosis*) in the alveolar macrophages. Direct lung delivery of the antibiotics could, thus, bring advantages over conventional oral therapy, as it co-localises drugs and pathogenic agents in the same compartment. Inhalable carriers have, however, to be optimised to reach the alveoli, where macrophages are located. 

According to the World Health Organization (WHO), combined therapy of tuberculosis is mandatory to potentiate the treatment efficiency [5], and isoniazid (INH) and rifabutin (RFB) are two of the used first-line drugs [6]. They display different characteristics, namely regarding molecular weight and aqueous affinity. In this regard, INH is a 137 g/mol hydrophilic molecule, while RFB has 847 g/mol and is hydrophobic, thus requiring organic or acidic solvents to dissolve. In parallel, the literature reports that the use of different solvents in the spray-drying of individual drugs has an effect on particle size, density, shape and surface appearance. Specifically, it is described that hydrophilic drugs processed by spray-drying may generate smaller particle sizes if surrounded by solvents with higher water content [7]. In turn, hydrophobic drugs lead to smaller and more porous particles when processed in more organic solvents [8]. To our knowledge, the comparison of the effect of different solvents in a mixture of two drugs of opposite character (one hydrophilic and the other hydrophobic) in the same formulation is not reported.

Bearing in mind the usefulness of a single formulation of inhalable microparticles conjugating two antitubercular drugs, following the WHO requirements on tuberculosis therapy, this work focused on studying the effect of using different co-solvents in the spray-drying process. Specifically, ethanol and hydrochloric acid (HCl) were tested as co-solvents, along with water, in a spray-drying process envisaging the production of ChS microparticles loaded with INH and RFB. The molecular mass distribution was evaluated for ChS and ChS-based microparticles, and a rheological study performed, in order to determine the effect of spray-drying on the polymer properties. The effect of the microencapsulation process on the antimicrobial activity of the drugs was also evaluated, encompassing the effect of using different solvents. Finally, the respirability of the produced dry powders was characterised to ascertain the effect of the solvents on the aerodynamic properties, and to establish the potential of the carriers for an inhalable strategy to treat tuberculosis. 

## 2. Materials and Methods 

### 2.1. Materials

ChS was acquired from Creative Biomart (Shirley, NY, USA). Acetic acid, INH, Tween 80^®^, phosphate buffer saline (PBS) tablets pH 7.4, thiazolyl blue tetrazolium bromide (MTT), dimethyl sulfoxide (DMSO) and HCl were purchased from Sigma-Aldrich (Darmstadt, Germany). RFB was supplied by Chemos (Altdorf, Germany). Middlebrook 7H9 (M7H9) and oleic acid, albumin, dextrose and catalase (OADC) supplement were purchased from Remel (Lenexa, KS, USA). Ultrapure water (Milli-Q, Millipore, Watford, UK) was used throughout. All other chemicals were reagent grade.

### 2.2. Polymer Purification

ChS from Creative Biomart was not of pharmaceutical grade and contained 5.52% (*w*/*w*) of protein on its composition. A purification of ChS was, thus, performed by ethanol precipitation. To do so, commercial ChS was solubilised in water at 5% (*w/w*, 200 mL) and poured over 250 mL of ethanol. This was left to rest at 4 °C overnight. After that, the dispersion was centrifuged (22,000 g, 4 °C, 1 h). The polymer was recollected from the pellet, freeze-dried and stored in a desiccator for further use. All the experiments described below were performed with purified ChS.

### 2.3. Microparticle Production

ChS microparticles were prepared by spray-drying, either unloaded or containing an association of the antitubercular drugs INH and RFB. ChS was used at 2% (*w/v*) in all cases and the drug-loaded microparticles were produced at ChS/INH/RFB mass ratio of 10/1/0.5. While INH was solubilised in water, the hydrophobic character of RFB required the use of co-solvents. Ethanol 70% (*v/v*) and HCl 0.01 M were tested for this end and the obtained RFB solution was then added to the previously formed ChS/INH solution. When ethanol was used, the final water/ethanol ratio of 80/20 (*v/v*) was applied in the spraying solution, while HCl was used at a final concentration of 0.002 M. 

Microparticles were produced from both solutions using a laboratory mini spray-dryer (Büchi B-290, Büchi Labortechnik AG, Flawil, Switzerland) operating in open mode and equipped with a high-performance cyclone. The operating parameters were: inlet temperature: 175 ± 2 °C; aspirator setting: 90%; feed rate: 0.7 ± 0.1 mL/min; and spray flow rate: 473 L/h. These conditions resulted in an outlet temperature of 110 ± 2 °C. After spray-drying, microparticles were collected, placed in a dark flask and stored inside a desiccator until further use. 

The spray-drying yield was calculated by gravimetry, comparing the total amount of solids initially added with the resultant weight of collected microspheres.

### 2.4. Characterisation of ChS-Based Microparticles

The morphology of microparticles was characterised by field emission scanning electron microscopy (FESEM; FESEM Ultra Plus, Zeiss, Oberkochen, Germany). Dry powders were placed onto metal plates and 5 nm thick iridium film was sputter-coated (model Q150T S/E/ES, Quorum Technologies, Laughton, UK) on the samples before viewing. 

Microparticle size was estimated as the Feret’s diameter and was directly determined by optical microscopy (Microscope TR 500, VWR international, Leuven, Belgium) from the manual measurement of 300 microparticles (*n* = 3).

The particle size distribution was determined by laser light scattering (Spraytec, Malvern Panalytical, Malvern, UK). To do so, approximately 15 mg of dry powder was dispersed in 15 mL of 2-propanol and sonicated for 5 min. The volume-based size distribution was characterised and the particle sizes below which 10%, 50% and 90% of the spray lies determined, being expressed as Dv (10), Dv (50) and Dv (90). From these values, span was calculated as follows:

(1) $$\mathrm{Span}\;=\;\frac{Dv\left(90\right)-Dv\left(10\right)}{Dv\left(50\right)}$$

The analyses were carried out in triplicate with an obscuration threshold of 10%.

Tap density (g/cm^3^) was determined using a tap density tester (Densipro 250410, Deyman, Santiago de Compostela, Spain) by measuring the volume of a known weight of powder before and after tapping, respectively (*n* = 3). The determination of tap density involved tapping the sample until no further reduction of powder volume was observed (average of 180 taps). 

### 2.5. Determination of Drug Association and Release 

To determine the drug content, drug-loaded microparticles were incubated in 0.1 M acetic acid, under magnetic stirring for 30 min, thus ensuring complete dissolution of microparticles. After filtration (0.45 µm), quantification was performed by high-performance liquid chromatography (HPLC, Agilent 1100 series, Waldbronn, Germany). A LiChrospher^®^ 100 RP-18 (5 µm) column of 4 mm i.d. × 250 mm length with security guard cartridge (Merck, Darmstadt, Germany) was used. Detection was performed by a diode array detector at 275 nm. Mobile phase consisted of a mixture of 20 mM phosphate buffer pH = 4 (A) and acetonitrile (B), flowing at a rate of 1.0 mL/min. The gradient started as 95:5 (A:B) for the first 5 min and changed to 30:70 at 8 min, remaining as such until 14 min. It returned then to initial conditions at 16 min, for a total run time of 17 min. Under these conditions, retention times of INH and RFB were 4.0 and 12.0 min, respectively. A linear calibration plot for INH and RFB was obtained over the range of 10–400 μg/mL (*n* = 3). Drug association efficiency (AE) and microparticle (MP) loading capacity (LC) were estimated as follows (*n* = 3):

AE (%) = (Real amount of drug on MP/ Theoretical amount of drug on MP) × 100(2)

LC (%) = (Real amount of drug on MP/Weight of MP) × 100(3)

Drug release was determined in PBS pH 7.4 added with 1% (*v/v*) Tween 80^®^. The assay respected sink conditions, with the maximum amount of drug being always below 30% of maximum solubility [9]. INH solubility was considered 274.0 ± 4.8 mg/mL [10], while that of RFB was 0.496 mg/mL [11]. A determined amount of microparticles (30 mg) was incubated with the medium (10 mL), at 37 °C, under mild shaking (100 rpm, orbital shaker OS 20, Biosan, Latvia). Samples (1 mL) were periodically collected and the amount of each drug quantified as indicated above (*n* = 3). Similarity factor (f2) was used to compare the release profile of the different drugs and formulations (similar profiles present an f2 value not lower than 50) [12]. 

### 2.6. Molecular Mass Distribution

High-performance size-exclusion chromatography (HPSEC) was selected to define the molar mass profile of ChS, both in the form of polymer and microparticles (unloaded). To perform the analyses, the HPLC equipment was supplied with two columns (300 × 7.8 mm) in series (TSKGel G3000PWXL and TSKGel G2500PWXL, Tosoh Bioscience, Stuttgart, Germany), along with a PWX-guard column (40 × 6 mm). Samples were filtered (0.45 µm) and analysed using a refractive index (RI) detector, under the following conditions: 70 °C, Milli-Q water as mobile phase and flow rate of 0.4 mL/min. Dextrans (DX) with a molecular weight ranging from 12 to 80 kg/mol (Fluka, Buchs, Switzerland) were used as standards. Analyses were performed at least in duplicate.

### 2.7. Rheology: Steady-State Shear Measurements 

A rheological study was performed on ChS polymer, unloaded ChS microparticles and ChS/INH/RFB microparticles prepared with either ethanol or HCl. Aqueous dispersions at 10 g/L were prepared in all cases, dispersing the corresponding amount of sample in distilled water, under stirring for 15 min, at room temperature. Steady shear flow curves in terms of apparent viscosity vs. shear rate were conducted on a controlled-stress rheometer (MCR 302, Paar Physica, Graz, Austria) using a plate–plate geometry (25 mm diameter, 0.5 mm gap). Flow measurements were obtained by decreasing and, then, increasing shear rate, following a logarithmic ramp to assess the presence of hysteresis. All trials were made at 25 °C and were controlled by a Peltier system (±0.01). In all cases, aqueous dispersions were sealed with light paraffin oil to avoid water loss during measurements and were rested for 10 min in the measurement system to allow sample structural equilibration. All experiments were performed at least in triplicate.

### 2.8. Aerodynamic Characterisation of Microparticles Using an Andersen Cascade Impactor

Hydroxypropylmethylcellulose (HPMC) size 3 capsules (Capsugel, Colmar, France) were filled with 30 mg of ChS/INH/RFB dry powder, either prepared with ethanol or HCl. The content of three capsules was discharged in each aerodynamic test using the medium resistance RS01^®^ inhaler (Plastiape Spa, Osnago, Italy). The experiments were performed in triplicate. The device was connected to the Andersen cascade impactor (ACI, Copley Scientific, Nottingham, UK) which operates at 60 L/min, ensuring a pressure drop of 4 kPa through the device. This was activated for 4 s in order to let 4 L of air passing through the system, thus complying with the standard procedure described by USP 38 and Ph.Eur.8 [13,14]. 

ACI separates particles according to their aerodynamic diameter and it was assembled using the appropriate adaptor kit for the 60 L/min air flow test. Cut-offs of the stages (−1 to 6) at the air flow rates adopted in this work are reported in Table 1. A glass fibre filter (Whatman, Rodano, Italy) was placed right below stage 6 to collect particles with a diameter lower than that of stage 6 cut-off.

The plates of the impactor were coated with a thin layer of ethanol containing 1% (*w/v*) Tween 20^®^ to prevent particle bounce. The drugs were recovered from the apparatus with water/acetonitrile mixture (50/50, *v/v*) and quantified by HPLC (Agilent 1200 series), as described above. 

The quantification of drugs deposited inside the impactor enabled the calculation of different aerodynamic parameters. The emitted dose (ED) is the amount of drug ex-device, considered the total amount of drug collected in the impactor (induction port, stages −1 to 6 and filter). The metered dose (MD) is the amount of drug loaded in the capsules, determined by adding all the drug collected in the impactor and in the inhaler (device, induction port, stages −1 to 6 and filter). The mass median aerodynamic diameter (MMAD) was determined by plotting the cumulative percentage of mass less than the stated aerodynamic diameter on probability scale versus aerodynamic diameter on a logarithmic scale. The fine particle dose (FPD) corresponds to the mass of drug particles with an aerodynamic diameter lower than 5 µm calculated using the particle size distribution equation obtained from the ACI analysis. The fine particle fraction (FPF) is the ratio between FPD and MD.

The recovery (%) is the percentage of MD versus the labelled dose. The recovery ranged within 86% and 91% in all the experiments, being thus coincident with the requisites of the pharmacopoeia [13,14]. 

### 2.9. In Vitro Determination of Antitubercular Activity

The in vitro antitubercular efficacy of microparticles was evaluated against *Mycobacterium bovis* bacillus Calmette–Guérin (BCG). The Minimum Inhibitory Concentration (MIC) value of free and microencapsulated INH and RFB was determined by the microdilution method [15]. ChS/INH/RFB microparticles and the powders of free drugs were exposed to UV light for 10 min to provide sterilisation. Then, a solution of each dry powder was prepared at 1 mg/mL and diluted to the desired drug concentrations. First, 1 mg/mL stock solution of INH was prepared by dissolving the drug in the M7H9 supplemented medium. RFB was previously solubilised in DMSO (1 mg/mL) and then diluted with M7H9 broth. Drug stock solutions were mixed to reach concentrations corresponding to drug loadings. Two-fold dilutions of the antibiotics/formulations were performed to obtain final concentrations of RFB from 0.001 to 0.125 µg/mL and of INH from 0.008 to 1 µg/mL. A 1/10 dilution of a McFarland 1.0 turbidity standard suspension of M. bovis BCG was inoculated (20 µL of inoculum in 180 µL of medium or test solution) with a multichannel pipette, delivering approximately 10^4^ CFU per well. The outside row of wells (frame-like) were filled with sterile distilled water to avoid the evaporation of microplate content. The plates were covered with the lid, sealed with parafilm and incubated at 37 °C (Binder, Bohemia, NY, USA) for 7 days. Afterwards, 30 μL of MTT sterile solution were added to each well, followed by 4 h of incubation at 37 °C. Then, 50 μL of DMSO were added into the wells to solubilise the tetrazolium blue crystals that were produced, which is proportional to the growth rate of mycobacteria. The absorbance was measured by spectrophotometry (Infinite M200, Tecan, Salzburg, Austria) at 540 nm. The assays were performed in triplicate. The MIC value was considered the lowest that inhibited mycobacterial growth by 95%–100% [16,17].

### 2.10. Statistical Analysis 

The Student’s *t*-test and the one-way analysis of variance (ANOVA) with the pairwise multiple comparison procedures (Holm–Sidak method) were performed to compare two or multiple groups. All analyses were run using Sigmaplot (version 12.5) and differences were considered to be significant at a level of *p* < 0.05.

## 3. Results and Discussion

### 3.1. Preparation and Characterisation of ChS Microparticles

ChS underwent an initial step of purification in order to maximise polymer content and avoid the presence of proteins. The purified polymer was used to prepare microparticles loaded with a combination of INH and RFB. As far as we know, this is the first time that ChS is being proposed as a platform for the inhalable therapy of tuberculosis. The microparticles were prepared by spray-drying, resulting in yields of approximately 85% (Table 2), which were deemed very satisfactory [18]. INH is a hydrophilic drug, but RFB has hydrophobic character, thus requiring solvents other than water to solubilise. Despite spray-drying permitting to process a suspension, not requiring the solubilisation of all components of the spraying dispersion, it was decided to provide the complete solubilisation of both drugs in order to ensure a higher level of homogeneity in the final product. Ethanol and HCl were, then, tested as co-solvents of RFB, being mixed with water. 

**Figure 1:**
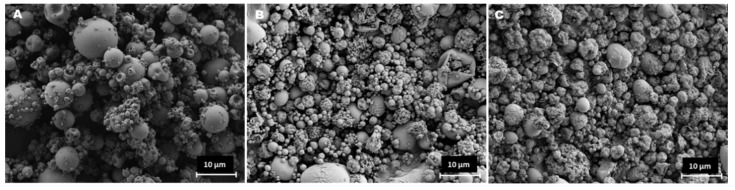
Microphotographs of unloaded chondroitin sulphate (ChS) microparticles (**A**) and ChS/ isoniazid (INH)/rifabutin (RFB) microparticles produced with water-ethanol (**B**) and water-HCl (**C**) as solvents, as obtained by scanning electron microscopy. Scale bar is 10 µm.

Disregard of the used solvent, the obtained particles generally exhibited a spherical shape, as observed in Figure 1. Moreover, the association of the drugs translated into the production of more wrinkled and corrugated microparticles (Figure 1B,C) comparing with the unloaded counterparts (Figure 1A). Objectively, the use of different solvents had no effect on microparticle morphology. The literature reports already several formulations of ChS microparticles, although none used spray-drying and different co-excipients were included in all cases. Nevertheless, it was interesting to notice that the spherical shapes and smooth surfaces observed in the unloaded microparticles reported herein were common characteristics [19,20,21]. The microphotographs suggest that the size of drug-loaded particles tends do decrease upon drug association, having a higher number of small particles comparing with unloaded particles. Comparing de Dv50 of the produced microparticles, a significant decrease from 9.6 μm of the unloaded particles to 4.1 μm of the drug-loaded counterparts was observed (*p* < 0.05, Table 2). This size range is reported as adequate to potentiate phagocytosis by macrophages [22], which could be advantageous in the treatment of intracellular diseases or vaccination approaches [23,24].

The tap density was determined to be around 0.5–0.6 g/cm^3^ for the dry powders corresponding to unloaded microparticles and microparticles produced with HCl, decreasing to 0.3 g/cm^3^ for microparticles produced with ethanol. A lower density has been correlated with less cohesive powders and better flowability [25], comprising an obvious advantage. INH was associated with microparticles with an of efficiency around 95%, independently of the used solvent. However, although not very accentuated, a significant difference was observed in the RFB association, which was 59% when ethanol was used versus 67% when HCl was the solvent (*p* < 0.05). The loading capacity was 8.2% for INH, varying within 2.6% and 2.9% for RFB, values that compare with the theoretical 8.6% for INH and 4.3% for RFB.

**Figure 2:**
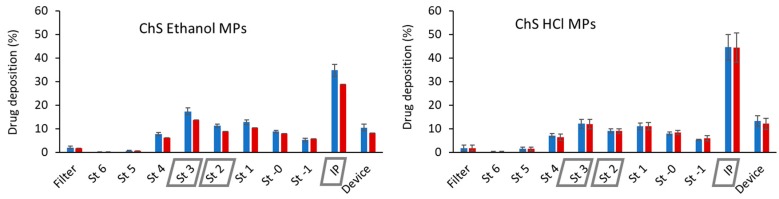
Stage-by-stage deposition profiles of isoniazid (blue) and rifabutin (red) inside the Andersen cascade impactor after aerosolisation of chondroitin sulphate (ChS) microparticles (MPs) with RS01 high resistance inhaler operated at 60 L/min (values are mean ± SD, *n* = 3). Grey boxes in the *x*-axis represent *p* < 0.05 comparing the same stage between the two formulations. IP: induction port; St: stage.

Figure 2 shows the stage-by-stage deposition profiles of both drugs encapsulated in the tested microparticles. The similarity of the profiles (INH vs. RFB) indicates that the two drugs were equally distributed in the various stages. This suggests the adequacy of spray-drying as a technique to produce microparticles with drug combination, resulting in a homogeneous composition independently of particle sizes, which led to co-deposition of drugs. A statistically significant difference in the deposition of both formulations was found in the induction port and in stages 2 and 3 (*p* < 0.05). This translated into differences in the calculated aerodynamic characteristics, which are displayed in Table 3. The dose emitted from the inhaler was very satisfactory in all cases, reaching 90%. This is indicative of the suitability of ChS to be used as microparticle matrix material in spray-drying, producing microparticles with good flowing capacity with any of the tested solvents. Similar MMAD values (3.8–4.0 μm) were obtained for both formulations. This aerodynamic diameter fits the range considered suitable to reach the respiratory zone, which is 1–5 µm [26,27], thus being adequate for a tuberculosis therapy approach. Moreover, FPF of 34%–44% was determined, indicating that this fraction of the microparticles has the aerodynamic characteristics for deep lung deposition. 

Despite having very similar MMAD, microparticles produced using ethanol as solvent provided a lower deposition in the induction port and higher deposition on stages 2 and 3, in the sequence of the determined higher FPD, enabling deep lung delivery of 1.5 mg of RFB and 3.1 mg of INH. The presence of ethanol apparently benefited the formulation, resulting in a less cohesive powder with better flowing properties. Despite the slightly lower drug association efficiency, the resulting aerodynamic properties provide increased drug accumulation in the respiratory zone comparing with that evidenced by microparticles produced with HCl. 

### 3.2. Impact of Spray-Drying on Polymer Characteristics

**Figure 3:**
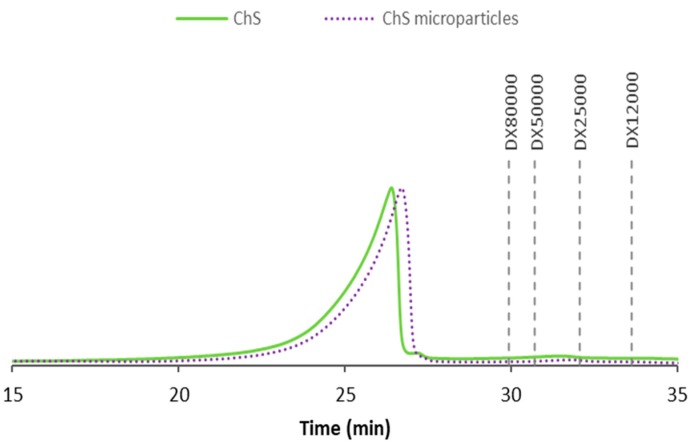
High-performance size-exclusion chromatography (HPSEC) profiles of chondroitin sulphate (ChS) as raw material, represented by the continuous line, and after processing by spray-drying (unloaded microparticles), illustrated by the dotted line.

Spray-drying was the technique selected to produce the microparticles as it permits tailoring their properties for deep lung delivery. Nevertheless, it was deemed important to study the impact of the process on the polymer properties. The molecular mass distribution of ChS was investigated as raw material and after the spray-drying process (unloaded microparticles), and the obtained profiles are exhibited in Figure 3. In both samples, the molecular mass was greater than 80 kDa, which was the highest standard available. The peaks of the two samples evidenced similar distribution profiles and, although showing a slight difference, the molecular mass was considered similar. Rani et al. (2017) isolated chondroitin sulphate from the cartilage of chicken keel bone and reported the peak of molecular mass at 100 kDa [28]. 

**Figure 4:**
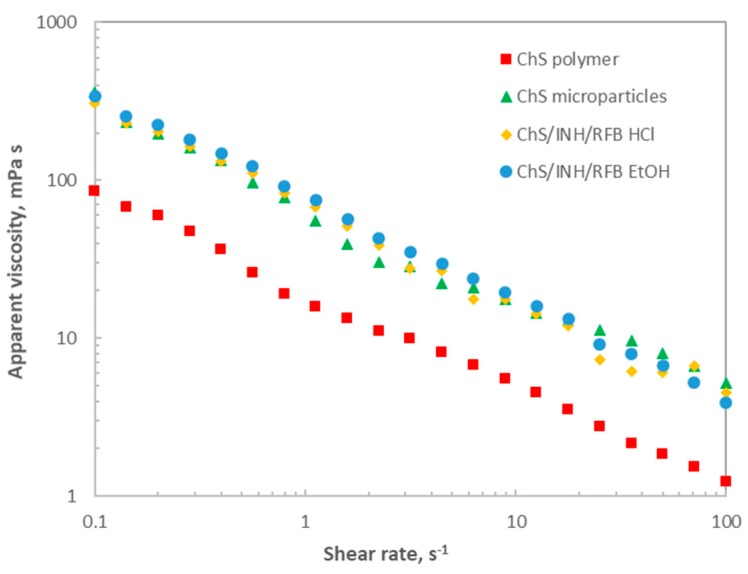
Steady shear flow curves for aqueous dispersions of chondroitin sulphate (ChS) polymer, ChS unloaded microparticles and ChS/INH/RFB microparticles produced with ethanol (EtOH) or HCl, at a polymer concentration of 10 g/L and 25 °C.

A rheological study was also performed, evaluating the rheological behaviour of the polymer before and after spray-drying and further determining the effect of associating the drugs. Figure 4 displays the steady shear flow curves of the tested aqueous dispersions (10 g/L) at 25 ºC. In all cases, shear-thinning behaviour was observed, with the apparent viscosity decreasing with increasing shear rate (about two decades). The apparent viscosity drop can be explained by the alignment of long chain molecules with each other at the highest shear rates, leading to easier flows. This behaviour is characteristic of pseudoplastic fluids, which may be described by the power law model, and the viscosity trend is consistent with a typical non-entangled polymer behaviour in the dilute regime [29]. At a fixed shear rate, it can be clearly observed that ChS polymer exhibited the lowest apparent viscosity over the tested range. After processing by spray-drying, higher apparent viscosity values were observed (about two-fold), suggesting an effect of the process on polymer characteristics. Since slight differences were identified in the HPSEC profiles, this behaviour suggests that some aggregates of the proper polymer chains may have formed during microparticle processing, which could involve higher flow resistance and, consequently, higher apparent viscosities. The samples corresponding to post-spray-drying products, all registered similar behaviour, indicating an absence of effect of both drug association and used solvents. Taking into account the envisaged application as inhalable tuberculosis therapy, this behaviour may help microparticles to maintain their “particulate” shape long enough to be phagocytosed prior to dissolution. It should be further highlighted that no hysteresis was observed in the tested samples, with the consequent advantage from the industrial point of view.

### 3.3. INH and RFB Release from ChS Microparticles

**Figure 5:**
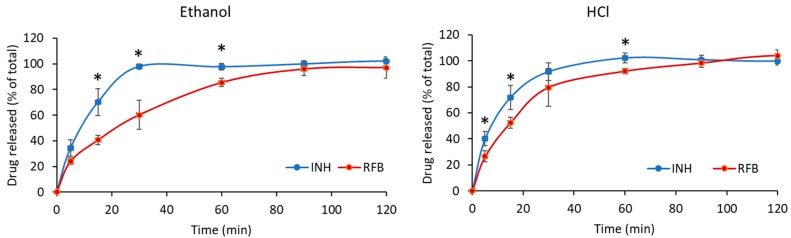
In vitro release of isoniazid (INH) and rifabutin (RFB) from ChS/INH/RFB (10/1/0.5, *w*/*w*) microparticles in PBS pH 7.4-Tween 80^®^, at 37 °C (mean ± SD, *n* = 3). * *p* < 0.05 comparing INH and RFB at a given time point.

Considering an application in tuberculosis therapy, drug release profiles were determined in PBS pH 7.4 added of 1% Tween 80^®^. The pH resembles that of the lung lining fluid [30] and the addition of Tween 80^®^ intends to mimic the surfactant content [31]. The results are displayed in Figure 5 and evidence rapid release of the drugs, as 100% release was registered within 60 min for INH and 120 min for RFB, in both formulations. The surface irregularities of microparticles, commented in Section 3.1, certainly contributed to the rapid release due to the increased contact with the release medium. Additionally, the high solubility of the polymeric matrix was another contributing factor, with the hydrophilic character of ChS resulting in rapid dissolution of the particle matrix and permitting a fast release of the drugs. Nevertheless, RFB tends to exhibit slower release than INH (*p* < 0.05). The similarity factor *f_2_* was used to compare the release of both drugs in each formulation and also the release of each drug in the two different formulations. The comparison between INH and RFB resulted in *f_2_* factor of 30 for microparticles produced with ethanol and 43 for microparticles produced with HCl. As *f_2_* values are lower than 50, it is concluded that differences exist in the release profiles of both drugs in both microparticle formulations. This is possibly a consequence of the higher molecular weight of RFB, along with its hydrophobicity, which contrasts with the hydrophilic character of INH. 

When comparing the release of each drug from both formulations, no differences were observed for INH, as this follows a similar release pattern from both microparticles, which is reinforced by an *f_2_* factor of 64. In contrast, Figure 5 evidences a difference in RFB release profile when the two microparticle formulations are compared. In this context, microparticles produced with ethanol provided somewhat slower release comparing with those produced with HCl (*p* < 0.05). An *f_2_* factor of 46 confirms the dissimilar profiles. Considering the results of the rheological study, no differences in the characteristics of the polymer were found comparing the two spray-dried microparticle formulations that could justify this behaviour. Therefore, one possible explanation for this effect could be that HCl remaining in the formulation after spray-drying facilitated RFB solubilisation afterwards, by providing a certain degree of protonation [32]. Another reason could be the existence of a different pattern of dispersion of RFB within the microparticle matrix in each formulation. If RFB is located more at the surface in microparticles produced with HCl, comparing with those produced with ethanol, this could justify the observed profiles. Nevertheless, further studies would be needed to study this possibility. 

The encapsulation of the same pair of drugs was reported in other works that used different microparticle matrix materials. The comparison of release profiles supports the conclusion that the matrix rules the release pattern. Although ChS is a hydrophilic polymer, it was able to provide a slower release of these drugs in comparison with microparticles composed of fucoidan, another hydrophilic polymer, which has shown a total release of INH and RFB in 30 min [33]. Locust bean gum, in turn, provides more viscous solutions and, thus, sustained the release of INH until 120 min and RFB up to 240 min [34], while low molecular weight chitosan microparticles released completely INH and RFB in 120 min [35]. Chitosan, but with higher molecular weight, provided more sustained release of INH, up to 40-60 h, which was demonstrated to be influenced by the polymer properties and crosslinking degree [36]. In fact, polymeric particles devoid of crosslinking agents typically show a faster release of the associated drugs [37].

It should be stressed that this assay does not mimic in vivo conditions, as it is well known that the alveolar epithelium is covered by a thin layer (~0.2 µm) of lung lining fluid [38]. Therefore, microparticles are expected to be only partially in contact with the fluid, and not immersed in it, as in this assay. Consequently, we anticipate that, in in vivo conditions, these microparticles will show slower drug release kinetics, allowing microparticle internalisation by macrophages before particle dissolution and complete drug release.

### 3.4. Antibacterial Activity of Drugs after Microencapsulation

One of the most commonly administered vaccines worldwide and the only one against tuberculosis is based on *M. bovis* BCG, a live attenuated vaccine. BCG vaccine is commonly used in experiments as a surrogate for virulent *M. tuberculosis* [39]. For this reason, and because BCG is a slow-growing strain with similarities to *M. tuberculosis*, it was selected to perform this assay, determining the MIC value of INH and RFB and the effect of spray-drying on this parameter. The MIC value of free and encapsulated drugs was determined by exposing *M. bovis* BCG during seven days to different concentrations of free drugs, association of free INH and RFB (at INH/RFB = 2/1, to mimic the drug ratio present in the microparticles) and to the two different microparticle formulations. The MIC value observed for the free drugs was 0.125 µg/mL for INH and 0.004 µg/mL for RFB, indicating a higher sensitivity of *M. bovis* to RFB than to INH. The literature reports variable values, depending on the bacterial strains and determination methods that are used, but the attained values were similar to those reported in other studies [39,40]. When a combination of both drugs was analysed, RFB ruled the inhibition effect and the same 0.004 µg/mL concentration (0.004 RFB and 0.008 INH) led to inhibition of the growth of *M. bovis*. Drug-loaded microparticles evidenced the same MIC value observed for the free drugs in combination, disregard of the formulation. This provides a dual indication: (1) that the spray-drying process does not affect the antitubercular effect of the drugs and (2) that the used solvent has no effect on this property of microparticles.

## 4. Conclusions

ChS microparticles were able to successfully associate a combination of INH and RFB, with efficiency between 60% and 95%, either using ethanol or HCl as co-solvent in the spray-drying process. The microencapsulation process induced some alterations in the rheological properties of the polymer, which was independent of the used solvent. In any case, the antitubercular efficacy of the drugs remained unchanged after encapsulation. Despite both the produced formulations of microparticles revealed similar aerodynamic properties and in vitro respirability, the dry powder prepared using ethanol as co-solvent showed a better ability to sustain the release of RFB. Regarding the objective of associating INH and RFB, two drugs of opposite aqueous affinity, in the same formulation of ChS microparticles aimed at lung delivery, the use of ethanol as co-solvent was considered to be advantageous.

## Figures and Tables

**Table 1:** Cut-off aerodynamic diameter (µm) for stages of Andersen cascade impactor (ACI) used at 60 L/min.

Stage-1	Stage-0	Stage 1	Stage 2	Stage 3	Stage 4	Stage 5	Stage 6
8.60	6.50	4.40	3.20	1.90	1.20	0.55	0.26

**Table 2:** Production yield (PY), median volume particle size (Dv50), tap density, drug association efficiency (AE) and loading capacity (LC) of ChS microparticles (mean ± SD, *n* = 3). Different letters indicate statistically significant difference within the same parameter (*p* < 0.05).

Microparticles	PY (%)	Dv50 (µm)	Span	Tap density (g/cm^3^)	AE%	LC%
ChS	73.3 ± 4.4^a^	9.6 ± 0.2^c^	2.0 ± 0.0^e^	0.50 ± 0.01^g^	n. a.	n. a.
ChS/INH/RFB (ethanol)	83.4 ± 1.0^b^	4.1 ± 0.1^d^	2.9 ± 0.1^f^	0.32 ± 0.03^h^	INH: 94.9 ± 5.7^j^	INH: 8.2 ± 0.5^m^
					RFB: 59.0 ± 6.9^k^	RFB: 2.6 ± 0.3^n^
ChS/INH/RFB (HCl)	84.9 ± 1.1^b^	4.1 ± 0.0^d^	3.1 ± 0.1^f^	0.58 ± 0.02^i^	INH: 94.6 ± 4.0^j^	INH: 8.2 ± 0.3^m^
					RFB: 67.6 ± 1.4^l^	RFB: 2.9 ± 0.1^o^

AE: association efficiency, ChS: chondroitin sulphate, INH: isoniazid, LC: loading capacity, PY: production yield, RFB: rifabutin, n.a.: not applicable.

**Table 3:** Aerodynamic characteristics of ChS/INH/RFB (10/1/0.5, w/w) microparticles (mean ± SD, *n* = 3). Different letters indicate statistically significant difference within the same parameter (*p* < 0.05).

Microparticles	Powder Emitted Dose (%)	Drug	MMAD (µm)	GSD (µm)	FPD (mg)	FPF (%)
ChS/INH/RFB (ethanol)	90.9 ± 1.0^a^	INH	3.8 ± 0.1^b^	1.9 ± 0.1^c^	3.1 ± 0.3^d^	43.7 ± 2.4^g^
		RFB	3.9 ± 0.1^ b^	2.0 ± 0.1^c^	1.5 ± 0.1^e^	42.6 ± 1.7^g^
ChS/INH/RFB (HCl)	89.6 ± 1.8^a^	INH	4.0 ± 0.2^ b^	2.0 ± 0.0^c^	2.9 ± 0.1^d^	35.0 ± 1.7^h^
		RFB	4.0 ± 0.3^ b^	2.1 ± 0.1^c^	1.2 ± 0.1^f^	34.0 ± 3.7^h^

FPD: fine particle dose; FPF: fine particle fraction; GSD: geometric standard deviation; MMAD: mass median aerodynamic diameter.
